# Perspectives: Evaluation of Older Adult Cochlear Implant Candidates for Fall Risk in a Developing Country Setting

**DOI:** 10.3389/fneur.2021.678773

**Published:** 2021-05-26

**Authors:** Christine Rogers

**Affiliations:** Department of Health and Rehabilitation Sciences, Faculty of Health Sciences, University of Cape Town, Cape Town, South Africa

**Keywords:** cochlear implants, developing countries, falls, older adults, vestibular deficits

## Abstract

Dizziness, vertigo, and falls are common in older adults. Data suggest that cochlear implant candidates are no different and could be argued to be at elevated risk due to the presence of hearing loss and likely vestibular involvement. *Perspectives* contextualizes current testing and screening paradigms for vestibular deficits and fall risk and suggests a protocol suitable for use in developing country settings.

## Introduction

**Figure 1:**
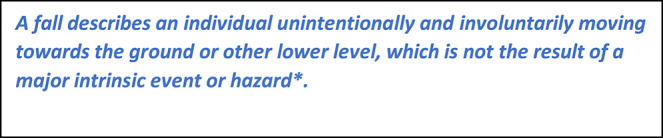
Suggested definition for a fall. ^*^Intrinsic fall risk factors are idiosyncratic health-related issues, such as visual acuity or balance deficits and may include age, sex, and ethnicity (12). Loss of consciousness due to syncope or stroke is an example of a major intrinsic event excluded from this definition of a fall (13, 14). The definition excludes major extrinsic events such as pedestrian traffic accidents (15).

Falls are common events in older adults with one in four falling each year (1). There is little reason to suspect the narrative is any different in emerging regions and, in fact, could be worse as “old age” and its attendant health-related problems may start as early as the end of the reproductive years (2). While more than 400 risk factors for falls exist (3), usually intrinsic and extrinsic factors combine with disease and aging to make falls and their adverse sequelae a reality for many. One important risk factor for falls is the presence of dizziness and vertigo, which are common complaints in older adults (4) and, along with subjective imbalance, increase the odds ratio 12-fold (5). Abundant studies describe the anatomical and physiological impact of the aging vestibular system although gaps in the literature exist. Scanty literature discusses the functional impact of vestibular impairment on daily living (6) as well as the delineation of the exact relationship between vestibular impairment, aging, and falls (7). Furthermore, review of the literature concerning falls is confounded by operational and methodological issues, for example, how a fall is defined. Differences in definitions for falls obfuscate the generalizability of clinical trials, treatment strategies, and outcome evaluation, including meta-analyses (8, 9). Researchers are urged to use a standardized definition of falls, such as the one promulgated by the Prevention of Falls Network Europe (PRoFaNE), which has been widely adopted by the scientific community (10, 11). Thus, when considering protocols for evaluation of patient groups, the definition shown in Figure 1 is recommended for researchers and clinicians alike. The definition concurs with recommendations from the American and British Geriatric Societies, the World Health Organization, and the UK National Institute for Clinical Excellence (NICE) (13).

Hearing loss is another risk factor identified as strongly correlated with fall events. One meta-analysis demonstrates that the presence of audiometrically proven hearing loss resulted in an almost seven-fold increased risk of falling (16). Further correlates of hearing impairment in older adults relevant to fall risk include sedentary behavior, slower gait speed, social isolation and withdrawal, and cognitive decline, itself a risk factor for falls (17–19). Cochlear implant (CI) candidates tend to have severe-to-profound hearing loss, which is likely to increase risk fall risk further. CI candidates may well have associated vestibular loss as the etiology of the loss could have affected both cochlear and vestibular apparatus. Around half of CI recipients are thought to have abnormal vestibular function prior to implantation although the procedure itself may cause temporary or permanent damage (20).

It is reasonable to assume that vestibular lesions before and after implantation extend to elevated fall risk. For example, one small study (20 participants, mean age 52 years, range 27–78 years) suggests impaired postural control in individuals' pre-implant assessments and cites a higher risk of falling (21). Participants underwent a battery of tests that evaluated sway using a mobile posturograph. Many of the tests resembled functional activities of daily living, such as walking with added head movements, short walks, and transitioning from sitting to standing. Participants' scores were compared with sex and age-matched normative data. Using the Vertiguard equipment, fall risk is regarded as scores of ≥40%. Preimplantation, fall risk in the CI group had a mean of 51% (range 24–80%) compared with normative data of 0–40% risk. The comparatively low mean age of the CI candidates is notable. Although this was an underpowered study using equipment not in common use, the strength is the choice of static and dynamic balance activities that were challenging for CI candidates. At the very least the study signals a need to consider fall risk in adult CI recipients. Rather than using a form of posturography, Stevens and coworkers (2014) (22) used the modified Clinical Test of Sensory Integration of Balance (m-CTSIB) (see later in this article) to assess their patients before and two weeks after CI. Nine of the 16 participants experienced a statistically significant decline (signaling poorer balance performance) in m-CTSIB scores post-operatively. Although the controversy regarding the links between vestibular function and deficits on the m-CTSIB must be acknowledged (22, 23), nevertheless, individuals over 60 years of age had a relative risk for falls of 2.1, more than their younger counterparts.

Another small study (24) evaluated the presence of vertigo in CI candidates pre-and postoperatively. Prior to implantation, half the participants had vertigo with abnormality in instrumented tests, including calorics and VEMP (see later in this article). More than one in three (36%) patients reported balance impairment postoperatively. Of pertinence to this *Perspectives* article, older adults, especially those over 75 years of age, were more likely to have long-term impaired vestibular function, which the authors (24) suggest was a sign of fragility and risk of falls. Interestingly, Colin et al. (24) and Louza et al. (21), and Amin et al. (25), suggest either an overall improvement in balance in some patients post-CI or, at least, no increase in the rate of injurious falls.

Falls have a detrimental and long-term impact on quality of life (26, 27) and are life-changing events for older adults (28). There is compelling evidence that the health status of adults who fall, in terms of physical, cognitive, and mental function, is fundamentally different from older adults who do not fall (29). Moreover, mortality linked to injurious falls is a serious concern. Evidence suggests that death rates from falls have risen precipitously in the last decade (30, 31). The WHO estimated 646,000 fall-related adult deaths each year; 80% of which occur in low- and middle-income countries (32). Older adults are particularly susceptible with most fall-related deaths recorded in individuals over 65 years of age (32). Frequently, survivors of the immediate postfall period have guarded outcomes in terms of both morbidity and mortality. Older adults are at increased risk for head, neck, and pelvis injuries compared with their younger counterparts (33). For example, falls are the leading cause of traumatic brain injury and are heavily implicated in hip fractures in older individuals (34, 35). It is not possible to overstate the devastating effects of an injurious fall for an older adult or the cost to public health budgets (36, 37). These concerns raise questions that all clinicians and researchers should be considering when dealing with CI programs. The studies discussed in this section should, at least, prompt consideration of obligations to older adults regarding potentially undesirable changes to vestibular and balance status post-implant. Is the patient at risk for falls? What is the best way to identify and manage that fall risk? What should comprise the minimum fall risk assessment, counseling, and safety precautions before and after an invasive procedure that may impair vestibular function at least temporarily? Implementation and evaluation of a protocol to explore vestibular deficits and fall risk requires further research.

Contributors to vestibular lesions pre- and post-implantation and, thus, elevated fall risk in CI candidates are briefly discussed next.

### Vestibular Lesions Pre- and Post-implantation

CI candidates are not a homogenous group, and causes of hearing loss may well be associated with progressive vestibular deficits. Examples include Ménière's disease, vestibulotoxicity, and ossification of the labyrinth post-meningitis. Older adults may have presbyvestibulopathy in addition to their cause for hearing loss. Presbyvestibulopathy is a chronic vestibular syndrome characterized by unsteadiness; impaired gait; falls; and mild, bilateral vestibular deficits on specialized investigation (38). The term “presbyvestibulopathy” supercedes others, such as presbyvertigo, presbyastasis, and presbyataxia (39). Ibrahim et al. (40) describe several potential mechanisms for vestibular deficits linked to the CI surgery itself. Mechanisms include trauma induced by electrode insertion, serous labyrinthitis due to the cochleostomy, a foreign body reaction labyrinthitis, endolymphatic hydrops, and finally electrical stimulation from the implant itself (40).

Symptoms associated with implantation may be episodic, delayed, or permanent and are thought to arise from the damage caused by CI, alteration of the vestibular receptors, and/or possible effects on the central nervous system (41). Postoperative complaints of vertigo are thought to be common although the incidence appears to vary widely (41). Clinicians who only question patients regarding vertigo *per se* may miss reports of unsteadiness, imbalance, instability, and dysequilibrium as well as falls. A meta-analysis by Hänsel et al. (41) suggests that vertigo was found in 16.8% of adult patients post CI, and as expected, a marked age effect was demonstrated. Age at implant was a significant risk factor with an age threshold of 59 years thought to herald increased risk, a finding supported by other authors. Again, variability in the incidence of postoperative symptoms is noted with results from Ibrahim et al. (40) suggesting approximately one third of recipients reported dizziness post-implantation. Importantly in terms of fall risk, the time for recovery, and possibility for incomplete recovery (compensation) increases for individuals over the age of 70 years (42).

## Specialized Equipment-Based Assessment of Vestibular Function in Older Adult CI Candidates

Over the last two decades, more extensive testing of the vestibular pathway has become more available in the clinic, leading to greater diagnostic accuracy (43). All five of the vestibular end organs can be evaluated given the appropriate equipment. However, specialized equipment is less available in under-resourced settings, so alternative screening strategies are suggested later in this *Perspectives* article. Despite flourishing CI programs in some emerging regions and the likelihood of these being located in at least secondary or tertiary level facilities, the specialized equipment and testing discussed in the following section could be out of reach. For example, in the Western Cape of South Africa, which has approximately seven million citizens [83% of all South Africans are reliant on state healthcare services (44)], only one tertiary facility has limited objective tests (VNG, VEMP, vHIT) available. The center at which most CIs are performed has no equipment. Another province has three implant centers and no vestibular apparatus whatsoever. South African Cochlear Implant Group guidelines suggest the use of the Dizziness Handicap Inventory and mentions calorics, vHIT, and C-VEMP (all discussed later in this section) as being suggested by the literature and in clinical use for bilateral or sequential CI procedures, but they stop short of mandating these measures (45).

Formal testing of vestibular function might guide decisions as to which ear to implant rather than solely relying on audiologic criteria (20). The ear with the vestibular deficit is likely the ear selected for implantation (46). Very little research reports preimplantation vestibular function screening, nor is there consensus as to the protocol for screening and management of the challenges associated with conducting assessments in CI patients (46). Furthermore, each test described in this section has distinct advantages and disadvantages in a CI population and may be influenced by non-vestibular issues including cooperation and alertness. Aging effects themselves are thought to have widespread but variable impact on the instrumented tests (47) described here. Hearing loss associated with visual impairment presents specific concerns in terms of vestibular assessment as do cognitive issues that may impact understanding instructions. Testing might be done with fixation abolished or in reduced lighting conditions, meaning that patients are unable to speech read or hear instructions to improve the quality of the results.

Caloric testing, usually as part of a videonystagmography (VNG) test battery, has been extensively researched (48) and is a mainstay of testing horizontal, semicircular canal function. VNG can also inform regarding the status of central vestibular and oculo-motor pathways, making identification of lesions therein possible (49). VNG offers advantages and disadvantages when applied in an older population. First, age has been linked to mild, progressive oculo-motor decline, which would show on the relevant subset of tests on VNG (47). Although central causes are thought to be present in around 25% of vertiginous patients in specialized facilities (50), oculo-motor deficits found on VNG should not result in exclusion from CI candidacy.

Caloric testing is not without its challenges. First, information regarding vestibular function is limited in that the stimulus is directed primarily at the horizontal semicircular canal (48). Although the calorics subtest is most useful to identify an asymmetry in responses between the two ears (5) as noted previously, presbyvestibulopathy may result in mild, bilateral loss of function to which calorics are relatively insensitive. However, in clear cases of asymmetry, guidance toward which ear to implant is possible. There are few studies on the impact of age on calorics, and age-related decline has not been empirically proven (47).

Patient-related concerns also have bearing on the results. Calorics may be uncomfortable and can induce symptoms of vertigo, nausea, and even vomiting. Symptoms may be so severe the patient declines further testing, leaving the battery of caloric tests incomplete. However, any temporary discomfort during testing is worth tolerating when compared with the risk of damaging the only ear with vestibular function during surgery. Furthermore, the impact of medication might influence the excitability of the responses and should be considered in an older population who often consume significant amounts of medication. Vestibular sedatives, in particular, might have a negative influence on results although opinion differs, and there is a lack of firm evidence (51). Finally, VNG is time-consuming, and post-VNG morbidity is a factor (52). An interesting point raised by Piker et al. (53) is that caloric testing may be influenced by changes in temporal bone anatomy post-CI and, thus, is not suitable for evaluating postoperative vestibular status. However, postoperatively, the focus should be on functional recovery.

Video head impulse testing (vHIT) is a newer addition to the armamentarium and is capable of assessing all three semicircular canals. vHIT assesses the gain of the vestibulo-ocular reflex. Vestibular gain is the ratio of slow-phase compensatory eye velocity to head impulse velocity (54). Abnormal responses suggest reduced gain in the canal under test (20), and a major advantage of the test is that each canal can be investigated separately. High specificity (which can be up to 100% depending on the extent of the lesion) (54) allows the potential “target” canals vulnerable to iatrogenic damage to be evaluated, obviating some of the issues with calorics restricted to testing the horizontal semicircular canal. vHIT is quick and easy to administer and well-tolerated (54). It takes time to practice the appropriate technique to optimize results, so the equipment cannot be regarded as “plug and play” (55). Patient-related factors that might make vHIT difficult to administer include those with a loss of facial skin tone, making the goggles too loose and issues affecting neck/head mobility (5, 46), such as arthritis. Systems with external cameras might be better for older populations with more appropriate management of artifacts and ill-fitting goggles. Changes with aging, which include reduced gain, still yield results within normal limits, making the test desirable (48). There are few studies that examine the impact of age on vHIT, but it appears that gain is stable up to the age of 70 years and then decreases and is most marked after the age of 79 (47). The portable nature of vHIT equipment (laptop and lightweight glasses with high-resolution cameras attached) makes vHIT intuitively appealing. Due to the inherent advantages of vHIT, which include ease of administration, acceptability to patients, and space and cost constraints, if only one piece of equipment were possible, then vHIT is a logical choice for under-resourced settings. Moreover, for CI centers with pediatric services, vHIT is far more acceptable to very young children (from 3 months) for whom calorics are not possible until the age of about 8 years (56). Therefore, combined with the results of bedside testing (oculo-motor tests, use of Frenzel lenses, and others) described in the next section, vHIT would feature strongly in a battery approach as a pass/fail criterion to identify CI candidates who require further evaluation and referral.

The final equipment-based test discussed here evaluates utricular and saccular function, viz., vestibular evoked myogenic potential testing (VEMP). VEMPs assess otolith function and the neural pathways (48). Two important patient-related variables are relevant for older adult CI assessments. Aging is a concern. The variability of the VEMP response increases with age to the point that the range is so variable and the yield so poor that certain authors suggest that there is little to be gained from conducting VEMPS in populations over the age of 70 years (38, 48). For example, the series by Piker et al. (57) demonstrates that, in participants with otherwise normal hearing and vestibular function, c-VEMPS were six times more likely to be absent in adults aged in their 50 and 60s, rising to 22 times more likely for adults over 70 years of age. Current practice guidelines (58) support the use of VEMP to diagnose semicircular canal dehiscence syndrome. Expert consensus holds that VEMP can be used to evaluate the extent of vestibular nerve involvement in vestibular deficits, but meta-analysis notes insufficient data for the efficacy of diagnosis of several specific vestibular disorders, including Ménière's disease (58). Standardization is required to increase the effective use of VEMP along with facilities developing their own data sets for both young and older patient cohorts (58). Therefore, at this time, the likely disadvantages of VEMP in older CI candidates outweigh advantages, such as speed and ease of administration.

Having discussed equipment-based tests, which might not be available in developing country contexts, a strategy for office-based clinical evaluation of CI candidates' vestibular and balance function is presented next.

## Low-Tech Assessment of Vestibular Function and Fall Risk Suitable for Emerging Countries

In developing regions, some consideration of either an office-based screening protocol or a system to select patients who should be referred for objective testing is necessary. Computerized testing for vestibular lesions, although more objective, is often costly, time-consuming, and demanding of space (59). Therefore, a more pragmatic approach is required that highlights the most sensitive and specific screening tests, which can be applied easily without the use of sophisticated and often expensive equipment. Selected tests should demonstrate clinical utility (ease and efficiency of use, resulting in relevant and clinically meaningful information) (60) and preferably be responsive so the effect of therapeutic interventions may be evaluated. The nature of screening tests implies that they could be conducted by several different cadres of staff, including audiologists, as part of the workup prior to CI. Mention must be made include the proliferation of tests available using fairly simple technology, such as laptops and smartphones, which is relatively inexpensive and required for a CI program in any case. Instrumented versions of tests such as Dynamic Visual Acuity are available for download to computers and in a virtual reality format. Mobile apps of the Subjective Visual Vertical test have been released at very little cost and are being evaluated for sensitivity and specificity (61–64). Commercially available interactive exergaming technology, such as the Wii Fit, is shown to give valuable and accurate information regarding balance control (65) and can be used for pre-habilitation and rehabilitation post-implantation.

## A Suggested Protocol for Vestibular and Balance Screening of Older Adult CI Candidates

The proposed protocol encompasses testing different constructs of vestibular and balance function. First, self-assessment scales or questionnaires are suggested. These instruments are often free from copyright and cost and can be completed at home, saving the clinician valuable time. Domains such as dizziness handicap, impact of symptoms on daily living, balance confidence, benefit from vestibular rehabilitation, and fall risk are explored in numerous well-constructed and validated scales, many of which are translated into major languages. An excellent resource is the Rehabilitation Measures database (https://www.sralab.org/rehabilitation-measures#our-database), which is a repository of measures commonly used in vestibular assessment and rehabilitation. Normative data and reviews of tests' psychometric properties are provided for many measures.

Key aspects of the case history are discussed next, followed by bedside tests. As the sensitivity and specificity of each clinical test varies, a test battery is helpful rather than singling out one or two tests. Finally, as discussed, aging increases the risk of falls, and vestibular deficits are a known risk factor for falls. However, vestibular inputs are just one source of information supporting the sense of balance. Balance requires the integration of signals from several systems, including vision and proprioception. Therefore, it is important to move the assessment beyond evaluation of vestibular end-organ function and to examine overall function and balance capacity along with fall risk (48).

### Self-Assessment Scales/Questionnaires

Two questionnaires are suggested: The first should evaluate the presence of symptoms of vestibular disorder, such as the short dizziness questionnaire from Roland et al. (66) or the Dizziness Symptom Profile (67). Colin et al. (24) propose a very simple, seven-item questionnaire for their CI series of patients, focused on the presence of vertigo and imbalance, quality of and associated symptoms, and timing. The brevity of the simplified Colin et al. assessment questionnaire is most appealing. As falls are such a concern in older adults, fear of falling and balance confidence should be assessed. The two most used scales, both of which have validated translations into many languages, are the Falls Efficacy Scale International (FES-I) and the Activities-Specific Balance Confidence (ABC). Generally, however, screening tools for falls perform poorly and are best used in conjunction with clinical judgement (68) and direct questioning regarding fall events, including slips, trips, and near misses. Of interest is the new fall risk calculator used for research, the FRAT-Up, into which patients' individual data can be entered, and a fall risk estimate is given on a dashboard (http://ffrat.farseeingresearch.eu/). The FRAT-Up correlates well with a history of falls (69). If the responses from the chosen questionnaires do not raise any concerns, potential CI candidates could exit the vestibular and falls assessment protocol at this point. Presence of a fall (whether injurious or not) within the last year should prompt further implementation of the suggested protocol.

### Case History

Case history is crucial! Although specialized and clinical testing may point to the site of a lesion, it should be acknowledged that there is little relationship between objective signs and the presence of symptoms due to central compensation processes. Thus, a case history is essential as is an assessment of self-perceived levels of handicap. The latter may indicate patients at risk for a poor prognosis in terms of functional recovery (70). Triggers and the temporal pattern of dizziness should be probed as these descriptions are more reliable than the type of dizziness described (71). The presence of associated symptoms may signal otological or neurological involvement. Routine medications should be reviewed and managed for their contributions to dizziness and fall risk, particularly instances of polypharmacy, which is increasingly frequent in older adults (72). Other causes for dizziness, particularly central causes, should be excluded. Patients whose history suggests progressive vestibular disorders, such as Ménière's disease, should be flagged for referral to a center with objective testing.

### Clinical Vestibular Screening Tests

Clinical tests of the vestibulo-ocular reflex include head thrust (also known as head impulse) testing, head shake, dynamic visual acuity, and hyperventilation. Using a test battery of screening vestibular tests enhances constructing a picture of unilateral or bilateral vestibular hypofunction and, thus, is recommended. As supported by vHIT, clinical screening for mild-to-moderate unilateral vestibular hypofunction is somewhat insensitive, so the head thrust test has limited usefulness for screening (73). However, the test is useful for identification of bilateral vestibular hypofunction (73) and is, thus, worthwhile conducting in a CI population. As with the instrumented test, technique is important (73). Patients identified with a positive head thrust should be referred for further testing, particularly if there is no acute cause for vertigo on the day of the test. Head shake performs more poorly than head thrust in terms of sensitivity but has good specificity (73). The test is commonly used despite poor evidence to support it, and of course, in patients with bilateral lesions, the test is even less helpful. Results are enhanced for tests such as head shake and hyperventilation if fixation is abolished, and cheap versions of Frenzel-type lenses are readily available.

The Subjective Visual Vertical (SVV) test can be done in an analog form or digitally using a mobile phone app, both in a bucket (64). Vestibular lesions are known to influence the perception of gravitational vertical. The test is quick and easy to administer, and the equipment can be assembled with little cost. Results are resistant to changes with age, making SVV appealing for an older adult population. Of interest for older CI candidates, some researchers suggest that the SVV can be helpful in the chronic phase of Ménière's disease (64) although, as with all the screening tests in this section, there have been questions regarding SVV's sensitivity and specificity (73). A recent meta-analysis has gone some way to refine the role of SVV in patients with peripheral vestibular disorder, and pooled results recommend SVV for the evaluation of vestibular function in patients undergoing vestibular surgery, such as vestibular schwannoma removal (74). Moreover, as discussed, VEMPs are not practical in an older and hearing-impaired population, so at least the clinical SVV gives some information regarding the otolith-ocular reflex.

As benign paroxysmal positional vertigo (BPPV) is so common, routine testing with a Dix-Hallpike maneuver and tests of the horizontal canal are highly recommended (73) along with appropriate treatment. CI candidates with a history of BPPV or new onset positionally induced symptoms should be screened after surgery to ensure that the condition has not arisen.

Screening tests might assist with lateralizing the side of lesion along with identifying possible bilateral lesions, and so can help refine the necessity for further testing in resource-constrained settings. Bedside evaluation may quickly answer questions as to which side to implant in unilateral recipients, but the role of vestibular compensation could influence the likelihood of positive findings. Finally, testing may help to identify patients who may need to be referred for vestibular rehabilitation therapy either before or after implantation. It is suggested that the following results, either in isolation or combination should trigger a referral for further testing: presence of spontaneous or gaze nystagmus, uni- or bilateral saccade/s on head thrust, abnormal SVV, nystagmus on headshake. Any BPPV should be treated and the patient reevaluated prior to further referral decisions.

### Exclude Another Common Condition: Orthostatic Hypotension

Orthostatic hypotension, with its associated dizziness and faintness on standing, can impair quality of life as well as reduce the ability to conduct the activities of daily living, making it potentially disabling (75). As orthostatic hypotension is linked to both dizziness and falls, clinicians working with older adults should be aware of the problem and the new diagnostic criteria from the Bárány Society (76) among others. Studies concerning the prevalence of orthostatic hypotension cite varying prevalence, likely linked to varying techniques for diagnosing the condition; however, a meta-analysis suggests that around 22.2% of older adults have the condition (75). This one in five prevalence makes a case for measuring blood pressure in supine and standing conditions in older adults.

### Tests of Static and Dynamic Balance

Tests of static and dynamic balance shift the focus from evaluation of the vestibular end-organs. Good balance is crucial for maintaining independence and competence in the activities of daily living along with preventing falls. Different components of balance are involved in maintaining either static (standing quietly) or dynamic (moving) balance, and it is important to test both aspects of postural control. Vestibular deficits are shown to increase the likelihood of falling during performance of simple dynamic balance tasks, such as transitioning from sitting to standing or changing body position (77). A plethora of tests exist across different age groups and medical conditions. One static and one dynamic test of balance is suggested for screening older adult CI candidates in emerging regions. Both tests are simple, in common use, and require minimal training. Should the results be abnormal, more focused tests should be considered (e.g., MiniBESTest) along with strategies to evaluate and manage fall risk. The Clinical Test of Sensory Integration of Balance (now referred to as the modified or m-CTSIB) is superior to the Romberg tests of old and can be used to evaluate the different inputs to balance (vision, vestibular, proprioception), giving important information for a therapeutic focus. The m-CTSIB is reliable and uses minimum equipment (78). Normative data for different age groups have been published recently (73). The test should be done with shoes removed and may be conducted with the feet together or apart (78).

Dynamic gait tests assess mobility walking and transitioning and are suitable to assess the functional status of older adults (79) along with fall risk. Specific to the older adult population, tests including transfers from sitting to standing are suggested. One of the most popular is the Timed-Up-and-Go (TUG), which is frequently used in both research and clinical contexts, including primary care in developed countries. Controversy exists regarding the cutoff at which fall risk can be reliably identified. The Centers for Disease Control's recommendation of 12 s (80) should be adopted. Normative data are available, which clearly show the relationship between sex and age with slowing of scores (81). Enhancements involving dual tasking (manual and cognitive conditions) help sharpen the test. Although a cognitive version has shown significant correlations with fall events (82), the dual-tasking mode of walking and counting might be challenging for patients with limited numeracy skills.

## Next Steps

With a dearth of reports on screening protocols for older CI candidates, formal research is required to evaluate protocols' efficiency and clinical utility for vestibular and fall risk assessments. The simple nature of the screening assessments suggested in this *Perspectives* article has inherent appeal. Should one piece of equipment be considered, vHIT makes the most prudent choice. The proposed protocol lends itself to be adopted by a variety of professionals in different contexts. The author calls for audiologists in particular to embrace their role assessing and indeed managing vestibular disorders in older adults in general as well as CI candidates, which should include judicious application of vestibular rehabilitation therapy and fall risk–reduction strategies.

## Data Availability Statement

The original contributions presented in the study are included in the article/supplementary material, further inquiries can be directed to the corresponding author.

## Author Contributions

The author confirms being the sole contributor of this work and has approved it for publication.

## Conflict of Interest

The author declares that the research was conducted in the absence of any commercial or financial relationships that could be construed as a potential conflict of interest.
